# Avoidance of reproductive conflict and the evolution of menopause in chimpanzees

**DOI:** 10.1098/rsos.250385

**Published:** 2025-06-25

**Authors:** Lauren C. White, Dieter Lukas, Kevin E. Langergraber, Linda Vigilant

**Affiliations:** ^1^ Department of Primate Behaviour and Evolution, Max-Planck-Institute for Evolutionary Anthropology, Leipzig, Germany; ^2^ Arthur Rylah Institute for Environmental Research, Department of Energy, Environment and Climate Action, Heidelberg, Victoria, Australia; ^3^ Department of Human Behaviour, Ecology & Culture, Max-Planck-Institute for Evolutionary Anthropology, Leipzig, Germany; ^4^ Institute of Human Origins, School of Human Evolution and Social Chance, Arizona State University, Tempe, AZ, USA

**Keywords:** menopause, reproductive conflict, chimpanzees, kinship dynamics, genomics, relatedness

## Abstract

The reproductive conflict hypothesis suggests menopause is rare in nature because it is only evolutionarily favoured in specific dispersal and mating systems. In social groups with local mating, shared resource competition and female-biased dispersal, an increase in a breeding female’s relatedness to her fellow community members with age is expected to favour late-life reproductive cessation as a response to intergenerational reproductive competition. Here, we use observational and genomic data from the Ngogo chimpanzee community to characterize kinship dynamics and investigate the potential role of reproductive conflict in explaining a recent report of menopause in this community. We first find that, as predicted by simple models, the combination of female dispersal and local breeding leads to age-specific increases in relatedness between female and male community members. Next, we use the observed kinship dynamics in inclusive fitness formulae to test whether reproductive cessation might have been selected for in chimpanzee females. We find that kinship dynamics measured within subgroups of the community, where competition is presumably most intense, favour the evolution of menopause beginning around age 40. This is consistent with patterns of age-related fertility declines observed in Ngogo, suggesting reproductive conflict may have contributed to the evolution of chimpanzee post-reproductive lifespans.

## 1. Introduction

Classical life-history theory predicts that organisms should maximize their fitness by reproducing until the end of life [1]. Contrary to this expectation, in some species—most famously humans—reproductive and somatic ageing are decoupled and females stop reproducing long before they die [2]. Besides humans, substantial post-reproductive lifespans (from here, termed menopause) have been found in only a handful of toothed whale species [3,4] and recently in one of our closest living relatives, chimpanzees (*Pan troglodytes*) [5]. While it is still unclear whether the observed phenomenon in chimpanzees directly matches that of human menopause, the data nevertheless revealed a sharp age-related decline in reproduction well before female chimpanzees die. This decline is puzzling, because in most other social species females experience long lives, invest heavily in their grand-offspring and yet reproduce until death [6,7].

The forces driving the infrequent evolution of menopause are a topic of long-standing debate among evolutionary biologists [2,8–10]. Non-adaptive perspectives posit that menopause is either a byproduct of recent increased lifespans, or a consequence of antagonistic pleiotropy, where traits that enhance early-life fertility incur costs to reproductive function later in life [11]. Adaptive explanations (which are not necessarily mutually exclusive) for the evolution of menopause emphasize that by ceasing reproduction, females may gain inclusive fitness benefits from enhancing the reproduction of their relatives. The ‘grandmother’ hypothesis emphasizes the kin-selected benefits arising out of providing food, knowledge or support to existing offspring or young grand-offspring, and thus enhancing their survival and reproductive output [10,12–14]. The ‘mother’ hypothesis additionally argues that high rates of mortality in childbirth at old age favour investment in existing offspring over continued reproduction [10]. Neither hypothesis explains why, if non-maternal care improves prospects of dependent young, it is the older females who forego reproduction, rather than younger females as is typical in cooperatively breeding species [15,16].

This conundrum is addressed by the other pre-eminent adaptive explanation for the evolution of menopause, the reproductive conflict hypothesis, which suggests that specific, uncommon demographic patterns will favour early reproductive cessation by females in response to intergenerational reproductive conflict [16,17]. This conflict arises when resources are primarily competed for within social groups, and hence one individual’s offspring production comes at the expense of other group members’ offspring production. When dispersal is limited and mating occurs non-locally or when dispersal is female-biased and mating occurs locally, the mean relatedness of adult females to males and group members in general (also termed ‘local relatedness’), is predicted to increase with age. Such dispersal and mating systems are rare in social species, with non-local mating and limited dispersal being typical of many toothed whales [18], while female-biased dispersal and local mating is typical of chimpanzees and may be the ancestral condition in humans (as suggested by the higher frequency of co-residence among same-sex close kin for males than females in human hunter–gatherers [19]). These conditions and the resultant patterns of local relatedness are expected to favour ‘harmful’ social actions (which negatively impact other group members) when a female is young and has few relatives in her group, and a switch to ‘helpful’ social actions (which benefit other group members) later in life when she lives with more descendants. Although many behaviours are likely influenced by age-specific changes in local relatedness [20], in the context of the evolution of menopause, the relevant social actions are female reproduction as a costly action that increases competition for local resources (harming) and reproductive cessation which reduces resource competition (helping; 16,17).

The sex- and age-specific changes in relatedness to other group members (termed kinship dynamics [20]); that are expected under particular dispersal and mating systems were formally defined in a set of formulae by Johnstone & Cant [16]. Recent studies found that these models can predict real-world kinship dynamics, finding that the magnitude and direction of kinship dynamics matches those observed using partial pedigrees and observational data from killer whales [21] and six other group-living mammal species featuring a variety of mating and dispersal systems [20].

Chimpanzees, the species we focus on here, are predicted to show kinship dynamics similar to those assumed for ancestral human populations [20]. Chimpanzees live in mixed-sex groups (termed ‘communities’) of up to 210 individuals that share a common territory. While males remain in the community in which they were born for their entire lives, females typically disperse from their natal community to join a new community at adolescence [22], where they will remain and reproduce for the rest of their lives [23]. A previous analysis of a single population of West African chimpanzees (*P. trogolodytes verus*) in Taï National Park, Ivory Coast, indicated that, as expected given the pattern of male philopatry, female dispersal and within community reproduction, female relatedness to male community members increased with age [20].

As in other social mammals, female chimpanzees within a community are in reproductive conflict because they each need scarce food resources to raise their offspring. Female chimpanzees engage in scramble competition over fruit, and the resulting aggression and spatial dispersion of females is linked to their reproductive success [24]. But, unlike other social mammals, due to habitual female dispersal and male philopatry, in chimpanzees, younger immigrant females are unlikely to be related to the offspring of other females in the community and are thus expected to not gain any inclusive fitness benefits from reproduction by other group members. In contrast, older females have a chance to be related to the offspring of the younger females because their sons might be the fathers of these offspring. These dynamics are expected to lead to reproductive cessation in older females as described in the reproductive conflict model [16]. There is little evidence or scope for grandmothering effects in chimpanzees as aged females generally live apart from their daughters and are unlikely to be able to identify their son’s offspring [25]. Similarly, the mother hypothesis does not seem relevant to chimpanzees, as childbirth complications are likely low due to small foetus size relative to their birth canals: reviews of the causes of mortality in both wild and captive chimpanzees do not mention any cases where death was due to complications around childbirth [26–29]. Thus, Wood *et al*. [5] suggested that reproductive conflict may be the primary mechanism driving extended post-reproductive lifespans in this species.

In this study, we use observational, genomic and demographic data from the Ngogo eastern chimpanzee (*P. t. schweinfurthii*) community to determine whether the reproductive conflict arising from kinship dynamics favours menopause and, if so, at what age. Ngogo is especially interesting as it is the first chimpanzee community for which demographic and hormonal data have revealed a substantial post-reproductive lifespan in this species, with menopause onset beginning around the age of 40 and a complete end to reproduction around age 50, as occurs in humans [5]. Ngogo is an exceptionally large community (more than 110 reproductively active individuals) and all members have been identified and regularly monitored since 2004 [25]. Menopause is absent or rare in other chimpanzee communities, where, in contrast to Ngogo, few females live past the age of 50. It is unclear whether menopause is actually a species-typical trait that has not been observed in other chimpanzee communities because of the negative impacts of humans on chimpanzee survivorship, particularly through disease, or whether menopause is only observed under the particularly favourable ecological conditions experienced at Ngogo, akin to the phenomenon of menopause observed in many primate species who are supplemented with food and medical care in captivity [25]. While the resolution of the question of whether or not menopause is an evolved, species-typical trait in chimpanzees will ultimately require more long-term chimpanzee demographic data from chimpanzee groups exhibiting ‘natural’ mortality profiles, a first step in this direction would be to determine whether or not adaptive models, such as the reproductive conflict hypothesis, predict the evolution of menopause in Ngogo chimpanzees.

Chimpanzees exhibit a fission–fusion social system [22] under which community members are rarely all found in the vicinity of one another, but rather associate in temporary parties of varying size, composition and duration. The tendency of some individuals to preferentially use similar portions of the territory results in subgroups of individuals who associate more frequently with each other than they do with other community members. Competition for reproduction limiting resources, such as fruit trees, may thus be more intense among individuals with higher levels of socio–spatial interaction [24,30–32], as has been claimed for the household versus community or village in humans [33–35]. At Ngogo, male–female pairs that show long-term tendencies to frequently associate are also more likely to reproduce [36]. In the context of the reproductive conflict model, we are interested in the identity and number of same and opposite-sex individuals with whom a female is typically able to compete or cooperate. Association data allow us to define these relevant subgroups and we therefore consider both the community and subgroup level when assessing kinship dynamics and the reproductive conflict hypothesis in chimpanzees.

The previous investigation of the kinship dynamics in chimpanzees [20] was based on the Taï Chimpanzee communities [37] that have experienced dramatic declines due to disease, poaching and other negative human impacts. Thus, this population may have an age and kinship structure that is not representative of long-term chimpanzee evolutionary history [38]. The data for the study also did not cover the full range of ages for both sexes. Furthermore, relatedness values within this population were based on pedigrees, which may produce inaccurate estimates of the relatedness of group members due to their shallow depth, uncertain or missing parentage links and unknown relationships among founder individuals [39]. Local relatedness is better estimated using genomic data, an approach that is rapidly becoming feasible even for wildlife populations sampled using non-invasively collected source materials of DNA, such as faeces [40–43]. Genomic data are available for 72−87% of all reproductively mature individuals present in the Ngogo community during our multi-year study period, providing exceptionally accurate estimates of genetic relatedness [42].

Here, we first use the specific social characteristics of the Ngogo community to derive the expected kinship dynamics among chimpanzees, using modularity analyses to detect how many individuals a female typically interacts with so that we can run analyses at both the level of the subgroup as well as the community. Next, we use genomic-based estimates of relatedness to empirically examine the sex- and age-specific changes in kinship of the Ngogo chimpanzees and compare these to the predicted dynamics. We then use an inclusive fitness model to assess whether the observed age-specific changes in local relatedness are predicted to lead to selection for menopause. Finally, we investigate whether the predicted timing for the onset of menopause matches the observed timing of reproductive cessation in Ngogo females.

## 2. Material and methods

### 2.1. Study community

The Ngogo community of chimpanzees occupies a territory of approximately 35 km^2^ in the mid-altitude rainforest of Kibale National Park, Uganda [44]. All members have been identified and regularly monitored since 2004 [25], and genomic (exome-sequence) data is available for 212 Ngogo individuals as well as 247 chimpanzees from other Kibale communities [42].

The ages of natal individuals born after monitoring began in 1995 are known to within 1 day to 3 months. Ages of older individuals are estimated based on their physical appearance, pedigree relationships to other individuals and/or behaviour. Immigrant females are assumed to be 13 years old when they arrive in Ngogo [5,25].

We confine our analyses to ‘reproductively mature’ individuals (or breeders *sensu* [16]). Females are classified as reproductively mature when they are 13 years old. Although younger females have been observed to conceive at Ngogo, since immigrant females are assigned the age 13 when they arrive in the community, we use 13 as the age of reproductive maturity here to avoid the youngest age ranges being biased towards natal females. Males are classified as reproductively mature when they are 10.5, as the youngest age at first conception observed for a male at Ngogo was approximately 10.8 years.

We chose 2008−2015 as our study period because it encompasses a time period when the proportion of reproductively mature individuals in the community with available genomic data is at least 70% [42], and it excludes more recent time periods when a respiratory disease outbreak caused many mortalities [45] and the first signs of an eventual community fission were observed [46].

Across our study period, the reproductively mature population size of Ngogo ranged from 96 to 119 individuals (mean = 111), the proportion of females ranged from 55 to 61% (mean = 58%) and the proportion of sequenced reproductively mature individuals ranged from 72% to 87% (mean = 80%; for these summaries, we considered only individuals that were alive and residing in Ngogo for at least 6 months of each year of our study period). A total of 212 Ngogo and 247 unhabituated individuals were successfully sequenced after screening of 3496 DNA extracts for sufficient DNA quantity and quality [42,43]. We consider our sample to be an unbiased representation of the community.

### 2.2. Defining subgroups

At 15 min intervals the identities of chimpanzees associating in the same party (defined here as sharing a common space, e.g. ≤50 m apart) as the focal individual were recorded during 1−13 h long focal observation sessions [30]. From these data, we calculated annual dyadic association indices using the simple-ratio method [47] implemented in the program SOCPROG (version 2.9) [48]. Because the data are based on focal follows, the chain-rule was not applied. Resultant indices range from 0 to 1 and represent an estimate of the proportion of time that two individuals spent together across each year of our study period. We then used these association indices to assign subgroup memberships based on maximizing within-cluster and minimising cross-cluster association indices using the method of Newman [49] implemented in SOCPROG. Association data were unavailable for 2009, and so this year is excluded from analyses involving subgroup assignment.

### 2.3. Johnstone & Cant’s formulae

Johnstone & Cant [16] derived two sets of general formulae to first predict age-related changes in local relatedness of group-living animals and second, to calculate the expected selective pressures on females of specific ages to ‘help’ or ‘harm’ all other group members through social action. In the context of the reproductive conflict hypothesis, these social actions are cessation of reproduction (helping by decreasing local competition) or continued reproduction (harming by increasing local competition).

The first set of formulae (appendix A of [16]) predicts how the mean relatedness (mean *r*) of females to all reproductively mature male and female group members (*rm* and *rf*, respectively) are expected to change as a female ages based on seven life-history parameters: the number of reproductively mature males and females in her social group (*nm* and *nf*), the male and female dispersal rates (*dm* and *df*), the sex-specific replacement parameters (i.e. the number of individuals dying and being replaced by either offspring or immigrants in the reproductively mature population each generation; *um* and *uf*) and the proportion of offspring fathered by local males (*m*).

The second set of formulae (appendix B of [16]) describes an inclusive fitness model based on the local relatedness values and the known life-history parameters. These formulae derive the range of individual costs and group impacts (*c/b*) across which a female’s social action would be selected for at different ages across her lifespan. Individual cost (*c*) is always positive, while group impact (*b*) can be positive (helping social action) or negative (harming social action). In the context of the reproductive conflict hypothesis, individual cost (*c*) is defined as the immediate loss of offspring for the focal female, and group impact (*b*) is defined as the immediate gain or loss of offspring for others in the group, conferred by the social action (i.e. continued or ceased reproduction). The model is multi-generational, with individuals competing for breeding positions to produce future offspring. In a social mammal exhibiting the typical pattern of male-biased dispersal and local mating, the mean relatedness of a female to other group members decreases with age, suggesting that any reproductive restraint (i.e. ‘helping’ of group members) would be favoured in younger rather than older females. In contrast, chimpanzees exhibit female-biased dispersal and local mating, and mean female relatedness to other group members is expected to increase with age, with ‘helping’ behaviour (i.e. reproductive cessation) thus expected to be favoured in older females [16].

We re-coded both sets of formulae in R (version 3.5.3) [50], and validated them by confirming we could recreate the results and figures presented by Johnstone & Cant [16]. Re-coded formulae are available at GitHub [51].

### 2.4. Predicted kinship dynamics

We used Johnstone & Cant’s [16] first formulae to derive theoretically expected kinship dynamics for Ngogo, using parameters based on the observed size and composition of the community in the study period and previously published data [25,42,52].

To derive expected age-specific values of local relatedness for Ngogo at the scale of the entire community, we define a group size of 110 reproductively mature individuals, of which 66 were female and 44 were male (*nf* = 66, *nm* = 44), a female dispersal rate of 50% (*df* = 0.5), a male dispersal rate of zero (*dm* = 0) and only local breeding (*m* = 1). These values are all based on empirical observations and data from the long-term Ngogo study (*nf, nm, dm, m* [25]; *df* [42]). We set 30 timesteps of 2.5 years to obtain a sufficient resolution across 75 years, the projected maximum lifespan of chimpanzees in this community [25]. During each time step, we assumed a replacement for both sexes representing random adult mortality and random replacement with immigrants or offspring such that individuals are expected to reach the mean lifespan observed in this community (*um* = *uf* = 0.076), derived as 2.5 (length of each time step) divided by 32.8 (mean lifespan for both females and males [25]).

To calculate the expectations for when local relatedness is considered at a smaller scale, we used a group size and sex ratio based on the empirically derived subgroups (electronic supplementary material, table S1). The only relevant subgroups for our models are those in which females are competing or cooperating with same *and* opposite-sex individuals, and so we exclude four single-sex subgroups (size range: 3−10 individuals). While modularity scores across years were close to, or slightly above, the 0.3 thresholds used to define partly modular societies [49] (electronic supplementary material, table S1), we acknowledge that these scores are relatively low and indicate that many interactions were occurring outside the subgroups identified. Regardless, association data provide our best estimates for subgroups across which competition is expected to be the most intense between members and is thus relevant to the reproductive conflict hypothesis.

The mean number of mixed-sex subgroups per year was 3.1 (range: 2−5), the mean size of the subgroups was 31 individuals (range: 3−54) and the mean proportion of females within each subgroup was 58% (range: 19−87%). For the analyses at the level of subgroup, we therefore defined group size of 30 reproductively mature individuals, of which 60% were female (*nf* = 18, *nm* = 12). As with the community scale analysis, we assumed equal replacement per each of the 2.5 years for both sexes (*um* = *uf* = 0.076) across 30 time steps (75 years). We do not have data-derived dispersal rates for these association-based subgroups, and we thus integrated information from other sources and studies to derive reasonable assumptions for these parameters at the subgroup scale. We assumed a low male dispersal rate as males preferentially associate spatially and socially with their mother and her associates (*dm* = 0.1 [36]). Conversely, we assumed a high female dispersal rate as half of all females at Ngogo migrate out of the community entirely when they reach sexual maturity, and those that remain are expected to disperse from their socio-spatial subgroup to avoid inbreeding with fathers and brothers (*df* = 0.9 [42]).

Although long-term associations, such as may occur from sharing a socio-spatial subgroup, increases the likelihood that a male and female will reproduce [36], the extent of extra-group mating across such neighbourhoods is uncertain. Here, we assumed only local breeding (*m* = 1) within subgroups for direct comparison to inferences at the community level but also explored the predicted kinship dynamics with lower rates of local breeding (*m* = 0.5) at the subgroup scale as a supplemental analysis.

### 2.5. Local relatedness

Pairwise genetic relatedness (genetic *r*) was estimated for all pairs of Ngogo individuals included in the exome-sequencing dataset as in [42]. Briefly, variant sites were identified and genotype likelihoods were calculated from exome-sequencing data for 212 Ngogo individuals and 247 unhabituated chimpanzees from the wider Kibale population using the ANGSD software suite [53]. After stringent filtering, we retained 2 56 481 variant sites, for which we calculated allele frequencies using a subset of individuals representing a random, high-quality sample of the Kibale chimpanzee population (*n* = 247). Genetic relatedness was then calculated based on allele sharing for all pairs of Ngogo individuals using individual genotype likelihoods, and the above-mentioned allele frequencies in the program ngsRelate (version 2) [54]. More details on the stringent filtering and validation checks performed on the exome-sequencing data and resultant relatedness estimates can be found in White *et al*. [42].

For each year, we then calculated the mean genetic relatedness (mean genetic *r*) of each reproductively mature female member of Ngogo to all other reproductively mature female and male members of the community that year. Individuals were considered co-residents of the Ngogo community for a particular year if their tenure in the community overlapped by at least 6 months of that year. The resulting community-scale dataset consisted of 65 females, each of which had two data points (mean relatedness to all reproductively mature female Ngogo members and mean relatedness to all reproductively mature males Ngogo members) for each year that she was present in Ngogo over the study period (range: 1−8 years). The total number of data points was 804.

We next considered local relatedness within subgroups of Ngogo chimpanzees. We calculated the mean relatedness of each female, within each year of the dataset, to all other reproductively mature females and males in her assigned subgroup. This dataset is reduced compared with the wider Ngogo dataset as association data were not available for 2009, and because some females did not have enough association data collected within some of the years, or were assigned to a small single-sex subgroup and were thus excluded. The resulting subgroup-scale dataset consisted of 59 females, each of which had two data points (mean relatedness to all reproductively mature female subgroup members and mean relatedness to all reproductively mature male subgroup members) for each year that she was present in Ngogo over the study period (1–7 years), resulting in a total of 536 datapoints.

### 2.6. Observed kinship dynamics

We used the community and subgroup scale datasets to model the mean relatedness of a female to her group members as a function of two main effects—age of the female and the sex of the individuals against which mean relatedness was calculated—and their interaction. We ran two models fit using the community dataset and subgroup dataset separately. Both models used beta regression generalized linear mixed models (GLMMs) because our outcome variable, mean relatedness, is bounded between zero and one [55]. We added a constant (0.00001) to all mean relatedness values for identifiability of model parameters [56]. Female identity was included as a varying effect to account for multiple measures per female across the years of the study period. The models were fit using the Hamiltonian Markov chain Monte Carlo (MCMC) engine R-Stan (version 2.19.2) [57], implemented using the *map2stan* function of the *rethinkin*g package (version 2.0) [58], in R (version 3.5.3) [50]. We used weakly regularizing priors (intercept: normal [mean = −2, s.d. = 1.5]; slopes: normal [mean = 0, s.d. = 1]) and a non-centred parameterization (Cholesky decomposition applied to the varying effect priors) to improve model efficiency [58]. We examined trace plots, the effective sample size, and $\hat{R}$ values after model fitting to ensure convergence and sampling efficiency. We present and plot the results of these models as mean posteriors and 89% credibility intervals (CI).

### 2.7. Inclusive fitness model

We then used the mean posterior from our models and the specified life-history parameters as above, to recalculate the ranges of individual cost and group benefits (*c/b*) across which helping or harming social action is expected to be selectively favoured across a female chimpanzee’s lifespan. Johnstone & Cant’s [16] second formula predicts inclusive fitness consequences for specific female ages that have been scaled by mean generation time, enabling cross-species comparisons. For example, age one in the theoretical model is equivalent to the mean parental age in the population (i.e. the mean generation time). Therefore, to enable comparison to predictions from Johnstone & Cant’s formula, we assumed an mean generation time for chimpanzees of 25 years [52] and scaled observed chimpanzee ages across which we calculated mean posterior values accordingly (i.e. by dividing ages by 25). We calculate the expected inclusive fitness dynamics at the community and the subgroup scale.

## 3. Results

### 3.1. Predicted kinship dynamics

Using Johnstone & Cant’s [16] first set of formulae which describes expected kinship dynamics, and the known or assumed demographic characteristics of the Ngogo community and subgroups, we predicted the sex- and age-specific mean relatedness of a Ngogo female to all other reproductively mature individuals in her social ‘group’ (i.e. either the Ngogo community or her particular subgroup).

**Figure 1.**
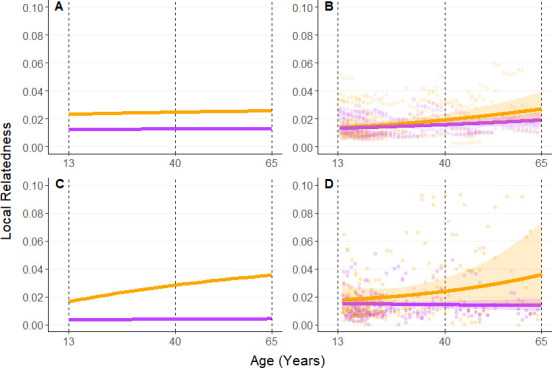
Age-specific changes in mean relatedness of reproductively mature females to all other reproductively mature females (purple) and males (orange) in her community (A and B) or subgroup (C and D). Theoretically predicted values (A and C) were derived using the formula of Johnstone & Cant [16]. Empirically derived values (B and D) are based on observed pairwise relatedness data fit in a beta regression GLMM with solid lines representing model posterior mean predictions and the shaded area representing 89% credibility intervals. Points represent the observed mean relatedness values for dyads involving females of a specific age. Vertical dashed lines show the age at which Ngogo females become reproductively mature (13 years) and show hormonal signs of menopause onset (40 years), and the age of the oldest female in our dataset (65 years).

As expected in a species with female dispersal, we found that a female’s mean relatedness to females in her social group was predicted to remain relatively stable over her lifespan (figure 1A,C, purple lines), and her mean relatedness to all reproductively mature males would increase with age (figure 1B,D, orange lines). Mean female relatedness to males was predicted to increase with age more strongly for the subgroup than community scale (figure 1C as compared with 1A, orange lines). At the community scale, the mean expected female relatedness to males increased from 0.023 at 13 years (reproductive maturity) to 0.026 at 65 years (the maximum female age during our study period; figure 1A), while mean expected relatedness to other female group members remained consistent at 0.013. At the subgroup scale, the female mean relatedness to males was 0.016 and more than doubled to 0.035 by the time she turns 65 (figure 1C). At the subgroup scale, changing the rate of local mating from 1.0 to 0.5 slightly reduced the relatedness overall, but the general pattern remained consistent (electronic supplementary material, figure S1).

Overall, the predicted kinship dynamics show that the relatedness of females to the males in their group is expected to be an order of magnitude larger than the relatedness of females to the other females, and that female relatedness to males is expected to increase across a female’s lifespan. This pattern persists across both the community and subgroup scales regardless of local breeding rates. However, the predicted increase in female-to-male relatedness across a female’s lifespan was greater when examined at the subgroup scale.

### 3.2. Observed kinship dynamics

Using our empirical data at the scale of the community, as predicted (figure 1A) we found that the mean relatedness of a Ngogo female to other reproductively mature individuals increased slightly as she aged (figure 1B; electronic supplementary material, table S2). At age 13 a Ngogo female’s mean relatedness to other reproductively mature community members, regardless of sex, was approximately 0.013 (figure 1B, males: mean = 0.014, 89% CI = 0.011−0.015; females: mean = 0.013, 89% CI = 0.011−0.015). At age 65 her mean relatedness to reproductively mature males in the community was 0.028 (mean = 0.028, 89% CI = 0.018−0.039) and her mean relatedness to reproductively mature females in the community was 0.019 (mean = 0.019, 89% CI = 0.014−0.024).

At the subgroup level, mean relatedness of a Ngogo female to other reproductively mature females in her subgroup was, as predicted, essentially unchanged as she aged (figure 1D, age 13: mean = 0.015, 89% CI = 0.012−0.017; age 65: mean = 0.014. 89% CI = 0.009−0.019). As expected, her mean relatedness to reproductively mature males in her subgroup increased (although there was also considerable uncertainty in the strength of this increase with the slope coefficient overlapping zero; figure 1D; electronic supplementary material, table S3). Mean relatedness to reproductively mature males in her subgroup at age 13 was 0.018 (mean = 0.018, 89% CU = 0.011−0.027), doubling to 0.037 at age 65 (mean = 0.037, 89% CI = 0.013−0.074).

Overall, we obtained reasonably consistent results when comparing predicted to empirically derived kinship patterns, pointing to the utility of the approach, although comparisons should be regarded with caution given the large confidence intervals of the empirical estimates. We note that at the community level, the theoretical model predicted a 13% increase in female relatedness to males over the reproductive lifespan, while a two-fold increase was observed in the empirical data (figure 1A,B). In contrast, at the subgroup level the predicted and empirical results were consistent in describing the magnitudes of the kinship dynamics (figure 1C,D). For example, female relatedness to other females was unchanged over the reproductive lifespan, while female relatedness to males doubled from ages 13 to 65, in both the predicted and empirical models. In sum, the subgroup-level data appear more consistent with the predictions of the model.

### 3.3. Empirically informed inclusive fitness model

We next used the mean posteriors from the empirical models of the observed kinship dynamics among the Ngogo chimpanzees above as relatedness values in Johnstone & Cant’s [16] inclusive fitness formulae.

**Figure 2.**
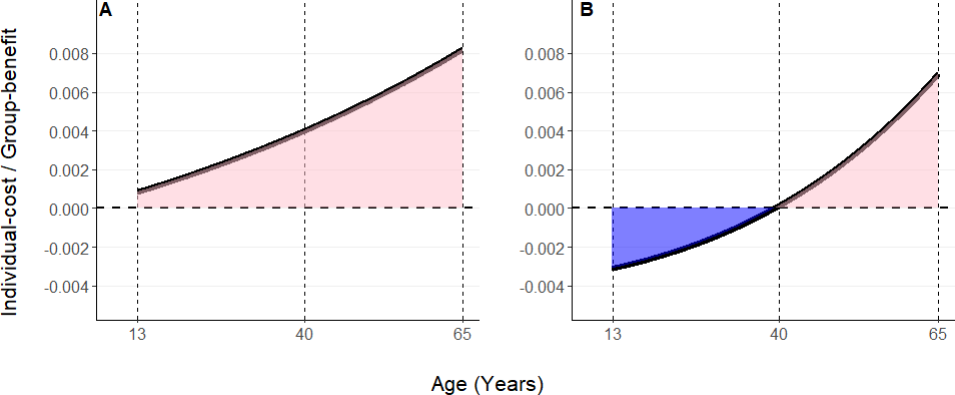
Selection on social behaviours given the observed kinship dynamics calculated within the community (A) or within subgroups (B). Harming social action by Ngogo females (i.e. reproduction) is expected to be favoured when the ratio of individual costs to group benefits is above the solid line, but below zero (blue). Conversely, helping social action by Ngogo females (such as reproductive cessation) is expected to be favoured when the ratio is below the solid line, but above zero (pink). Vertical dashed lines show the age that Ngogo females become reproductively mature (13) and show hormonal signs of menopause onset (40), and the oldest female in our dataset (65).

At both the community and subgroup levels an increase in helping with female age was observed. At the community level, helping social action was expected to be favoured across females’ entire lifespan, with the selective pressure increasing as they age (figure 2A). Conversely, at the subgroup level, harmful social action (i.e. reproduction) is expected to be favoured early in adult life and helping was expected to be favoured in later life, with the switch predicted to occur around at around 40 years of age (figure 2B). The rate of local mating is not part of the inclusive fitness model and therefore changes in this parameter do not further change these patterns.

## 4. Discussion

Our results show that the relatedness of female chimpanzees to males in their group increases as females get older, while their relatedness to other females in their group stays low across their life. These empirically derived kinship dynamics are visible at both the community and subgroup scale and are similar to the predicted values derived from Johnstone & Cant’s formula [16]. This confirms that, despite their simplicity, the dispersal and mating regimes encoded in the kinship formula are the key parameters to produce reasonable approximations of real-world phenomena, in agreement with previous studies [20,21,59]. Additionally, at the subgroup scale, we find that the empirically derived kinship dynamics and observed demography among Ngogo chimpanzees are expected to favour the evolution of post-reproductive life spans in females, beginning at around the age of 40. This is consistent with hormonal evidence and patterns of age-specific fertility from Ngogo chimpanzees, which suggest that female fertility declines sharply beginning in the early- to mid-40s before reproduction halts completely at around 50 years of age [5]. These results suggest that intergenerational reproductive conflict among closely associating females may have played a role in the evolution of menopause in chimpanzees.

We find that the observed social dynamics at Ngogo lead to age-related increases in the relatedness of female chimpanzees to other members of their social group, and that older females have a much higher relatedness to the males in the group than to the other females. The observed kinship dynamics matched the predictions from Johnstone & Cant’s models [16] reasonably well, with the results from the subgroup scale matching the predictions slightly more accurately, regardless of the assumed local mating rate. The slightly elevated relatedness observed at the community scale compared with predictions may be due to relatedness among females who have immigrated from the same adjacent communities, which the model, not being spatially informed, assumes are unrelated. Our community-scale relatedness patterns are consistent with those previously observed in a different chimpanzee community [20], suggesting common patterns across chimpanzee communities and populations.

The extent to which these relatedness patterns lead to the expected transition from competition to cooperation during the lifetime of a Ngogo female chimpanzee depended on the social scale of the group. Results from the subgroup scale support the reproductive conflict model of menopause evolution with harmful social actions expected to be favoured until around the age 40, when a transition to selection for helpful social actions is expected. Conversely, at the community level, positive social actions are expected to be favoured across a female’s lifespan and increasingly as females get older. The absolute values of average relatedness at Ngogo were low at both social scales (0.013−0.037); however, this is expected in large groups of social species [20,60]. As our inclusive fitness model demonstrates, even modest changes in average relatedness can significantly influence selective pressures over evolutionary timescales.

Since the observed kinship dynamics were similar at both community and subgroup scales, this difference in the inclusive fitness model results is attributable to the dispersal rates and group size parameters. We suggest that the female dispersal rate is the critical parameter here. Low female dispersal rates, as observed at the community scale at Ngogo [42], lead to a greater number of young females co-residing with kin in subsequent generations, increasing the inclusive fitness benefits of helping social actions at a young age. The outcome of the inclusive fitness model at the community scale predicts that commonly observed social helping actions in chimpanzees, such as coalitionary support, food sharing or grooming [61–64], should be more common among older females, which could be tested in future studies.

The most relevant scale of social organization in the context of the reproductive conflict hypothesis depends upon the scale at which resource sharing and competition between group members is most acute. For example, Johnstone & Cant [16] assumed that competition for resources within human societies would be concentrated within the ‘household’ or ‘extended family’ and thus considered the kinship dynamics of relatively small groups (number of adults = 6). The use of household as the relevant unit in humans is supported by studies that have documented negative impacts of reproductive conflict within household units [33–35]. For example, Lahdenperä et al. [34] found substantial reductions in the survival of offspring when the reproduction of daughter- and mother-in-laws overlapped, indicating that competition for resources within the household was intense. In chimpanzees, subgroups are hypothesized to form around core areas of food resources [18,32], suggesting that subgroups may be where competition for fitness-limiting resources is highest and reproductive conflict most acute. Future work could examine the magnitude of reproductive conflict in chimpanzees at different social scales by investigating the spatial distribution of resources and how well they are accessed by group members. This could be achieved, for example, by measuring feeding efficiency inside and outside of core areas or demonstrating the impacts of local competition by testing whether the number of infants born into a community or subgroup negatively correlates with juvenile survivorship.

The demography of the Ngogo subgroups is similar to those observed for other chimpanzee groups at the community level. The mean number of adult individuals in the Ngogo subgroups was 31, similar to the mean of 26 adults observed in 9 different communities [65]. It is important to note that the subgroupings used in the analyses here are based only on association and do not correspond to socio-spatial units termed western, eastern and central neighbourhoods previously described for the Ngogo community [46]. Indeed, the Ngogo community has split into separate communities since data collection for this study was completed [46], suggesting that smaller units may represent more demographically stable group sizes for chimpanzees. Taken together, this suggests that the kinship dynamics that we observe at the subgroup level may reflect those that are representative of chimpanzees more broadly, and this species’ long-term evolutionary history.

Our study does not address the question of whether extended post-reproductive lifespans are a universal feature in chimpanzees. Prior to the identification of menopause at Ngogo [5], previous studies of wild chimpanzee populations and communities did not find significant female post-reproductive lifespans [7,66]. There are two possible explanations for this apparent difference. One is that menopause is a universal trait in chimpanzees and extended post-reproductive lifespans were detected only at Ngogo because at other sites females die early from anthropogenic causes. In contrast to other communities, the Ngogo community has not experienced multiple rounds of epidemic disease and hence the adult lifespans at Ngogo may be more representative of chimpanzee evolutionary history than other study communities [25,67,68]. Notably, the data from the western chimpanzee (*P. t. verus*) communities at Tai did show a pattern of kinship dynamics consistent with predictions from the reproductive conflict model [20], suggesting that extended reproductive lifespans might be detected there if adults experienced longer survivorship.

The alternative explanation is that menopause is not a universal trait in chimpanzees. This idea invokes the substantial variation in group sizes, dispersal rates, predation, survivorship, fecundity and the availability of resources across chimpanzee sites [25,69,70] and suggests that this produced variation in kinship dynamics and potentially limited the selective pressure for prolonged female post-reproductive life. However, this hypothesis of menopause being selected for only in some chimpanzee populations requires that the populations have been sufficiently isolated to allow for differential local adaptation. It is unclear if that has been the case given genetic evidence for high levels of interconnectivity [71, 72], although local genetic adaptation, particularly to disease, has recently been documented [73]. It is noteworthy that genetically distinct populations of killer whales employing different foraging strategies (ecotypes) have similar qualitative but different quantitative kinship dynamics, and yet all have long post-reproductive female life spans. This suggests that this trait was present in their ancestral population more than 350 000 years ago [74] and may indicate limited plasticity in expression of this trait. Menopause is also ubiquitous in humans, even those living under exceptionally challenging circumstances [75] further suggesting that it is not a trait that varies flexibly.

While our analysis here focuses on the reproductive conflict hypothesis and benefits to group members arising from cessation of reproduction by post-reproductive females, if such females are present there is the possibility of other additional forms of kin-directed benefits. In killer whales, post-reproductive females appear to provide social support [76] and ecological knowledge regarding resources [2], increasing the survival of offspring [77] and even grand-offspring [14]. Studies on the potential benefits provided by post-menopausal females in chimpanzees are lacking due to the scarcity of such females. Nevertheless, is notable that in wild western chimpanzees, the males who experienced maternal death after weaning but before becoming mature had reduced mean reproductive success compared with males with surviving mothers [78]. In another study looking at the benefits of mothers, in bonobos (*P. paniscus*), the presence of a mother was shown to improve that male’s reproductive success [79]. Thus, it is possible that the patterns of dispersal and mating that lead to reproductive conflict and favour the evolution of female post-reproductive lifespans can also create conditions for further benefits arising out of the prolonged presence of the post-reproductive females.

If extended post-reproductive lifespans are a universal feature of all ‘natural’ chimpanzee populations, human menopause may be a much older trait than previously assumed, and universal and irreversible menopause in humans may even have evolved from the standing variation in post-reproductive lifespans within the *Pan–Homo* last common ancestor. Investigating and quantifying the factors, such as dispersal rates or household sizes that were relevant in the evolution of menopause in humans is exceedingly challenging using contemporary human populations [80, 59], although analyses of ancient DNA may allow some access to distribution of kin across geographical and temporal spans in the past [81]. Our results suggest that investigating the particular demographies, kinship dynamics and resource availabilities under which early reproductive cessation has been favoured across chimpanzee populations, along with consideration of the potential benefits provided by long-surviving mothers and grandmothers, will aid to understand potential drivers of the unusually long and pronounced post-reproductive lifespan in humans.

## 5. Conclusion

In this study, we have added to a growing body of research that has shown that kinship dynamics can be reasonably well predicted from dispersal and mating patterns using simple models. Furthermore, using empirically derived kinship dynamics, we show that under certain conditions extended post-reproductive lifespans will be adaptively favoured for chimpanzees, with the predicted timing of menopause onset matching the observed timing in Ngogo females at approximately 40 years of age. Finally, we have demonstrated that the scale at which kinship dynamics are considered and at which competition occurs has a large influence on the selective pressures for early reproductive cessation. The variability that chimpanzee populations show in extended post-reproductive lifespans and relevant life-history traits make them an ideal study system for investigating the selective pressures that have led to the distinctive human menopause.

## Data Availability

Code for reproducing Johnstone & Cant’s formulae as well as model code and input data are available at GitHub [51], and have been archived within the Zenodo repository [82]. For interested readers, we also provide a web-based application for exploring the impact of different demographic parameters on kinship dynamics and inclusive fitness, as predicted by Johnstone & Cant’s formulae (https://lozwhite.shinyapps.io/ShinyJohnstoneAndCant). Supplementary material is available online [83].
